# The Effects of Sesquiterpenes-Rich Extract of *Alpinia oxyphylla* Miq. on Amyloid-***β***-Induced Cognitive Impairment and Neuronal Abnormalities in the Cortex and Hippocampus of Mice

**DOI:** 10.1155/2014/451802

**Published:** 2014-08-07

**Authors:** Shao-Huai Shi, Xu Zhao, Bing Liu, Huan Li, Ai-Jing Liu, Bo Wu, Kai-Shun Bi, Ying Jia

**Affiliations:** ^1^Shenyang Key Laboratory of Active Components of Traditional Chinese Medicine Screening and Evaluation, School of Traditional Chinese Materia Medica, Shenyang Pharmaceutical University, Wenhua Road 103, Shenyang 110016, China; ^2^The Engineering Laboratory of National and Local Union of Quality Control for Traditional Chinese Medicine, School of Pharmacy, Shenyang Pharmaceutical University, Wenhua Road 103, Shenyang 110016, China

## Abstract

As a kind of medicine which can also be used as food, *Alpinia oxyphylla* Miq. has a long clinical history in China. A variety of studies demonstrated the significant neuroprotective activity effects of chloroform (CF) extract from the fruits of *Alpinia oxyphylla.* In order to further elucidate the possible mechanisms of CF extract which mainly contains sesquiterpenes with neuroprotection on the cognitive ability, mice were injected with A*β*_1−42_ and later with CF in this study. The results showed that the long-term treatment of CF enhanced the cognitive performances in behavior tests, increased activities of glutathione peroxidase (GSH-px) and decreased the level of malondialdehyde (MDA), acetylcholinesterase (AChE), and amyloid-*β* (A*β*), and reversed the activation of microglia, degeneration of neuronal acidophilia, and nuclear condensation in the cortex and hippocampus. These results demonstrate that CF ameliorates learning and memory deficits by attenuating oxidative stress and regulating the activation of microglia and degeneration of neuronal acidophilia to reinforce cholinergic functions.

## 1. Introduction

As the global population ages, Alzheimer's disease (AD) is rapidly becoming an urgent public health challenge. It has been estimated that the number of people with dementia in the worldwide was 35.6 million in 2010, and this number will almost double every 20 years, to 65.7 million in 2030 and 115.4 million in 2050 [1]. Therefore, searching for safe, better tolerated, and effective drugs is necessary.

AD is a progressive degenerative disease of the brain which is characterized by deterioration of memory and cognitive functions with formation of senile plaques and neurofibrillary tangles and the loss of synapses in the selected regions of the brain. Several hypotheses of AD, including the *β*-amyloid peptide (A*β*) cascade hypothesis and the cholinergic hypothesis, have been applied to investigate the etiology of AD [2]. Scientists have proposed the cholinergic hypothesis; namely, the alternations of cholinergic system are closely related to the damage of cognitive function and AD. Based on this theory, a number of studies have been launched on acetyl cholinesterase (AChE) inhibitor, and then AChE inhibitors have been successfully developed for clinical treatment of AD [3–5]. To eliminate the deposition of A*β*, the main research strategy includes the following two pathways: one is to search A*β* secretase inhibitors, mainly including *β*-secretase inhibitors; the other one is to prepare antibodies of A*β* by using the immunological method in order to reduce the deposition of A*β*.

A*β* is a 40–42 amino acid proteolytic fragment of amyloid precursor protein (APP). The cascade begins with the cleavage of the APP sequentially by *β*- and *γ*-secretase. The 42 amino acid A*β* fragment self-assembles into oligomers. Its oligomers have been considered as the principal toxic substances which induce oxidative stress, neuronal apoptosis, and increase of neuronal loss [6]. A*β*_1–42_ also has direct pharmacologic effects on synaptic function, impairing memory, and long-term potentiation in animal models [7]. Neurofibrillary tangle formation, oxidation, excitotoxicity, inflammation, synaptic compromise, demyelination, mitochondrial dysfunction, and neurodegeneration follow the interaction with oligomeric A*β* [8]. A*β* may also fibrillize to form insoluble aggregates that compose the neuritic plaques characteristic of AD. In rats treated with A*β*, the release of acetylcholine (ACh) and dopamine stimulated by nicotine decreased in the brain, which demonstrated the learning deficits observed in the A*β* protein-infused rats, is partly due to the impairment of neurotransmitter [9]. On the basis of the accumulating evidences on pathological roles of A*β* in the progress of AD, A*β*-injected animals have become a useful model for understanding the pathogeneses and progression of AD.

*Alpinia oxyphylla* Miq. is regarded as a precious drug and also a kind of condiment in Hainan district in southern China. Sesquiterpenes, diterpenes, flavonoids, and diarylheptanoids have been found in* Alpinia oxyphylla* Miq. previously and some of which showed inhibitory effect on nitric oxide (NO) production in lipopolysaccharide- (LPS-) activated mouse peritoneal macrophages [10–13]. There have been growing evidences showing that chloroform extract from the fruits of* Alpinia oxyphylla* possesses significant neuroprotective activity [14]. There are a few studies showed that* Alpinia oxyphylla* had therapeutic efficacy for senile dementia by reducing the apoptosis and free radical. However, the exact mechanism or components are not explicit [15]. On the basis of the findings above mentioned, we hypothesized that chloroform extract of* Alpinia oxyphylla* could ameliorate aging through inhibiting oxidative stress, improving the cholinergic system, and reducing A*β* levels in the brain. Therefore, in the present study, we investigated the actions of chloroform extract of* Alpinia oxyphylla* on cognitive ability, oxidative stress biomarkers, and A*β* deposition in the hippocampus and cortex of aging mice induced by A*β*_1–42_ to elucidate the underlying molecular mechanisms. Furthermore, compounds of the active fraction of the extract have been identified.

## 2. Materials and Methods

### 2.1. Material

*Alpinia oxyphylla* Miq. was purchased from Shenyang Tongrentang Drug Co., Ltd. (Shenyang, China). The crude drugs were of high quality and authenticated by Professor Ying Jia of Pharmacognosy Department, Shenyang Pharmaceutical University. Donepezil was supplied by Wanbang Pharmaceutical Company (Zhejiang, China). A*β*_1–42_ was obtained from Sigma-Aldrich (St Louis, MO, USA) and dissolved in sterile physiological saline (1.0 *μ*g/*μ*L) in the tube, which was then sealed and incubated for 120 h at 37°C to cause the peptide to aggregate. Commercial kits used for determination of AChE, GSH-px, MDA, *β*-secretase, and A*β*_1–42_ were purchased from Jiancheng Institute of Biotechnology (Nanjing, China) and Qiming Biotechnology Company (Shanghai, China).

### 2.2. Sample Extraction and Fractionation

The air-dried fruits of* Alpinia oxyphylla* Miq. (10.0 kg) were extracted three times for 2 h each time by refluxing in 95% ethanol (1 : 10, w/v). The filtrates were concentrated and dried in vacuum at 60°C. The crude extract was dissolved in distilled water and then partitioned sequentially in different solvents, namely, petroleum ether, chloroform (CF), ethyl acetate, and *n*-butanol, to fractionate the polar and nonpolar compounds in the crude extract. The resulting solvent fractions were concentrated by rotary evaporator and dried by a vacuum oven at 45°C. The doses of chloroform extract were expressed as gram of the original dry materials per kilogram body weight.

### 2.3. UPLC-ESI/MS Analysis for CF

The chemical composition of CF was analysed by using a Waters-UPLC-Q-TOF/MS with an ultraviolet/visible detector (UV/Vis) coupled to an ion trap mass spectrometer with an ESI interface. The chromatogram was recorded at 255 nm. An HSS T3 Column (100 mm × 2.1 mm, 1.8 *μ*m) with the column temperature set at 25°C was used for separation. The injection volume was 5 *μ*L, and elution was performed at a flow rate of 0.6 mL/min using a mixture consisting of acetonitrile (A) and 0.1% (v/v) formic acid (B). A gradient program was used as follows: 0–3 min, 20% A; 3–20 min, 20%–50% A; 20–25 min, 50%–90% A; 25-26 min, 90%–20% A; 26–28 min, 20% A.

Mass analyses were performed using an ESI interface in the positive ion mode. The data were acquired in the full scan and MS/MS^2^ scanning mode. The optimized instrumental parameters were set as follows: positive mode: desolvation temperature, 250°C; source temperature, 120°C; capillary, 3.0 kV; sampling cone 30.0; extraction cone 4.0; source temperature 130; desolvation temperature 450; desolvation gas flow (L/Hr) 800.0; collision energy 6.0 ev; and scan range,* m/z* 100–1000 amu.

### 2.4. Animals and Administration

**Figure 1:**
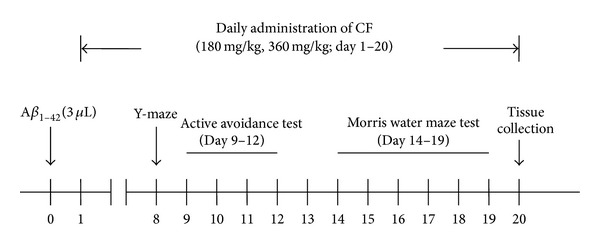
The experiment schedule.

Seventy-two male ICR mice weighing 18–22 g were provided by the Experimental Animal Center of Shenyang Pharmaceutical University (Shenyang, China). They were maintained on standard laboratory conditions of temperature 25 ± 1°C and a 12 h light/12 h dark cycle with food and water available ad libitum for the duration of the study. After 1 week of acclimatization, all mice were randomly divided into 6 groups (*n* = 12/group): vehicle control group, sham-operated group, model group, donepezil group, chloroform group 1 (CF1), and chloroform group 2 (CF2). All the mice were anesthetized with 3.5% chloral hydrate (0.1 mL/10 g). Then, model group, donepezil group, CF1 group (180 mg/kg), and CF2 (360 mg/kg) were injected with aggregated A*β*_1–42_ peptide (3 *μ*L) into the right lateral ventricle within 3 min by means of a stereotaxic apparatus (AP, −0.5 mm, ML, ±1.1 mm, DV, −3.0 mm). The needle was removed with 1 min delay to allow diffusion. Mice in the sham-operated group were injected in an identical manner with the same amount of physiological saline (3 *μ*L). From the next day, mice in the CF1 and CF2 were administered with CF of 180 mg/kg and 360 mg/kg in distilled water containing 1.0% DMSO (20 mL/kg, i.g.) daily for 20 consecutive days by intragastric infusion (i.g.). Mice in donepezil group were administered with DPZ (0.65 mg/kg, i.g.). Mice in vehicle control group, sham-operated group, and model group were treated with distilled water containing 1.0% DMSO (20 mL/kg, i.g.) in the same period. The experiment schedule is shown in Figure 1. Animal care was in accordance with the Guidelines for Animal Experimentation of Shenyang Pharmaceutical University and the protocol was approved by the Animal Ethics Committee of the institution.

### 2.5. Behavioral Experiments

#### 2.5.1. Y-Maze Test

Y-maze test was used as a measure of immediate spatial working memory which was a form of short-term memory [16]. Y-maze is a three-arm maze with equal angles between all arms. Mice were initially placed within one arm, and the sequence and number of arm entries were recorded manually for each mouse over an 8 min period. The alternation score (%) for each mouse was defined as the ratio of the actual number of alternations to the possible number (defined as the total number of arm entries minus two) multiplied by 100 as shown by the following equation: Alternation% = [(Number of alternations)/(Total arm entries −2)] × 100%. The number of arm entries was used as an indicator of locomotor activity.

#### 2.5.2. Active Avoidance Test

Learning and memory ability were detected by the active avoidance as described previously [17]. During the training session each trial began when the animal was introduced into any of the compartments with its head oriented toward the wall opposite to the mouse hole. After a variable period (±60 s), a conditioned stimulus was delivered. If the mouse crossed to the opposite compartment during the presence of the conditioned stimulus, an avoidance response was scored. If the mouse did not cross during the presence of the conditioned stimulus the unconditioned stimulus was delivered and remained on for 10 s or until the animal escaped to the opposite compartment. If the animals crossed to the opposite compartment within 10 s, an escape response was scored and a new trial began. The session consisted of 30 trials and it ended when 30 trials finished. The percent of conditioned avoidance response and total time of test were recorded.

#### 2.5.3. Morris Water Maze Test

Learning and memory ability were detected by Morris water maze test as described previously [18]. The experimental apparatus consisted of a circular water basin (150 cm in diameter, 60 cm in height), containing water (25 ± 2°C) to a depth of 40 cm, which was rendered opaque by adding black nontoxic carbon ink. A platform (9 cm in diameter, 38.5 cm in height) was submerged below the water surface and placed at the midpoint of one quadrant. It was given 90 s to find the platform and was allowed to rest on it for 15 s. The animals which failed to find the location within the given time were gently guided to the platform and were allowed to stay on it for 15 s; each mouse was given two trial sessions each day for five consecutive days, with an intertrial interval of about 15 min. To determine whether the animal would take a spatial learning strategy to locate the platform, a single spatial probe trial was assessed on day six, the platform was removed from the water basin, and the mice were allowed to swim freely for 60 s. All data were recorded and analyzed by a computerized video imaging analysis system (Huaibei Zhenghua biology apparatus Co., Ltd, Anhui, China).

### 2.6. Sample Preparation

After probe trial sessions of Morris water maze test, 72 mice were sacrificed by cervical dislocation and the brain was immediately removed. The cerebral cortex and hippocampus of 6 mice in each group were each dissected out [19]. The brain (except for the cerebellum) of the rest of the 6 mice in each group was also dissected out. Each part of the brain tissue was stored at −80°C until the biochemical studies [20]. Before detection, each part of the brain tissue was rapidly homogenized in ice-cold saline and the homogenates were centrifuged at 3500 rpm at 4°C for 15 min. The supernatant was collected for assay.

### 2.7. Assay of GSH-px and Lipid Peroxidation within the Brain of Mice

The left cerebral cortex tissue was homogenized in ice-cold saline and centrifuged at 2000 rpm for 10 min, and the supernatant was collected. The activities of GSH-px and the amount of MDA in the supernatant were measured using commercial assay kits.

### 2.8. Determination of AChE Activity in the Cerebral Cortex and Hippocampus of Mice

The activities of AChE were measured using colorimetric methods [21]. Briefly, the reaction mixture containing samples, DTNB, and sodium phosphate (1 mmol/L, pH 8.0) was preincubated for 10 min at 37°C, and then acetylthiocholine iodide was added to the reaction mixture to incubate for 5 min at 37°C. The absorbance was measured at 412 nm at room temperature. AChE activity was expressed as nmol/mg of protein.

### 2.9. Assay of *β*-Secretase in the Cerebral Cortex of Mice

The activities of *β*-secretase in cortical of mouse brain were measured using a specific *β*-secretase ELISA kit according to the manufacturers' protocols. This formation of fluorescence was read using a fluorescence plate reader with excitation at 335–355 nm and emission at 450 nm.

### 2.10. Determination of A*β*_1–42_ in Hippocampus of Mice

Each brain was homogenized in 8 vol. (w/v) of cold 5 mol/L guanidine HCl/50 mmol/L Tris-HCl and mixed at room temperature for 4 h. Dilutions of the extracts were made in Dulbecco's phosphate-buffered saline containing 5% BSA and 0.03% Tween 20 (pH 7.4) supplemented with 1x protease inhibitor cocktail (Roche, Germany). Following centrifugation at 16,000 ×g for 20 min at 4°C, aliquots were diluted with sample buffer provided by the manufacturer and used for the measurement of A*β*_1–42_ levels by enzyme-linked immunosorbent assay (ELISA) (IBL, Germany).

### 2.11. Histology

For the histological examination of tissue sections, hematoxylin-eosin and Congo red were used as described previously [22]. The brain was removed and kept overnight in the last fixative solution for dehydration. Then cut it into transparent slices and imbedded it in paraffin. Brain samples were cut into coronal sections. Serial sections were selected around the needle trace. Sections were stained with hematoxylin-eosin reagent and then dehydrated with graded alcohol and mounted with neutral balsam medium to observe changes in the cortical and hippocampal neurons.

### 2.12. Statistical Analysis

All values were expressed as the mean ± SD. Statistical differences in all groups were analyzed using one-way ANOVA. Student's *t*-test was used to determine significant differences between groups. Differences were considered statistically significant at a value of *P* < 0.05.

## 3. Results and Analysis

### 3.1. UPLC-ESI/MS Analysis for Chloroform Extract of* Alpinia oxyphylla*

**Figure 2:**
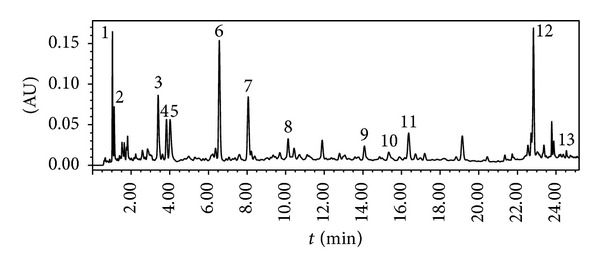
UPLC chromatogram of chloroform extract of* Alpinia oxyphylla* at 255 nm. 1, oxyphyllanene A; 2, protocatechuic acid; 4, 11S-nootkatone-11,12-diol; 5, 11R-nootkatone-11,12-diol; 6, teuhetenone A; 7, teuhetenone B; 11, oxyphyllol B; 12, Nootkatone; 13, dibutyl phthalate.

The chemical compositions in CF were analysed via UPLC-ESI/MS. The chromatogram of CF was obtained at 255 nm (Figure 2). The results of UPLC-ESI/MS and tentative identification are shown in Table 1. Among these compounds, some showed typical fragmentation patterns as previously reported, while the others were identified based on the retention times and the UV spectra of the reference standards [23, 24].

### 3.2. Y-Maze Test

**Figure 3:**
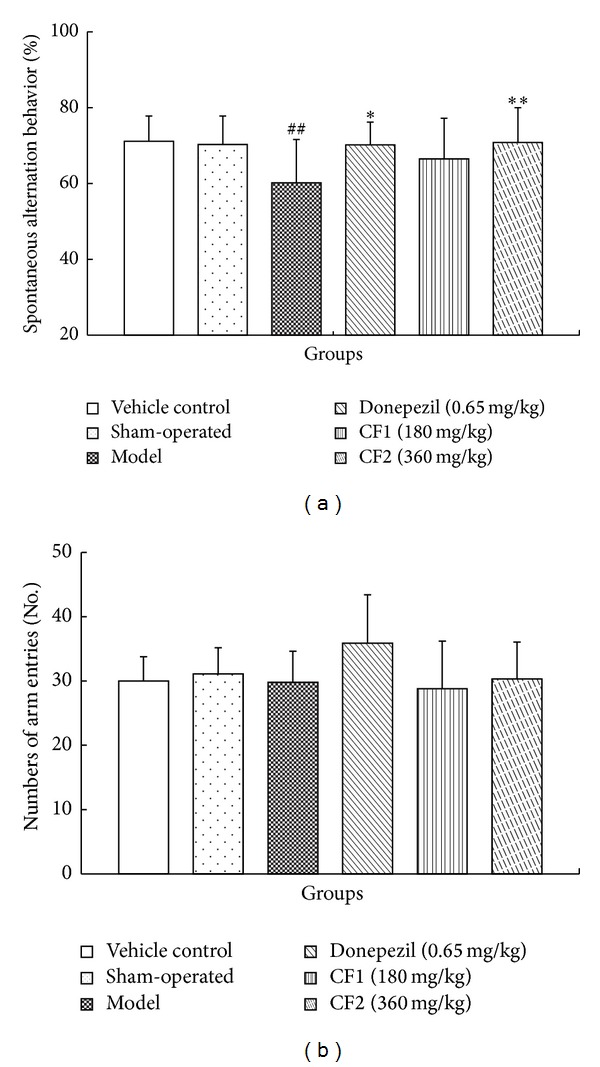
Effect of CF on A*β*_1–42_-induced memory deficits in the Y-maze test. Spontaneous alternation behavior (a) and the number of arm entries (b) during an 8 min session were measured. Data represent means ± SD (*n* = 10). ^#^ *P* < 0.05, compared with sham-operated group. *P < 0.05, **P < 0.01 compared with model group.

The effect of CF on short-term or working memory was investigated in the spontaneous alternation behavior Y-maze test. As presented in Figure 3(a), spontaneous alternation was significantly different between groups (*F* (5, 54) = 3.338, *P* < 0.05). The spontaneous alternation of model mice was significantly lower than that of sham-operated mice by 18% (*P* < 0.05), and the lowered spontaneous alternation induced by A*β*_1–42_ was significantly reversed by CF2 (360 mg/kg) by 17% (*P* < 0.01). Moreover, the effect of CF (360 mg/kg) on the spontaneous alternation behavior was similar to that of donepezil (*P* < 0.05, Figure 3(a)). However, numbers of arm entries were similar in all experimental groups, demonstrating that general locomotor activity was not affected by CF (Figure 3(b)).

### 3.3. Active Avoidance Test

We assessed the effects of CF on learning and memory ability in mice exposed to A*β*_1–42_ (3.0 *μ*g/mouse) using active avoidance test described above.  Table 2 showed that condition avoidance response percent every day and total time in four days but had no marked difference between the control and sham-operated groups. Compared with control group, the condition avoidance response (CR) was significantly decreased (day 2–4) in model group (d2,* F* (5, 54) = 4.289, *P* < 0.05; d3,* F* (5, 54) = 3.617, *P* < 0.01; d4,* F* (5, 54) = 4.471, *P* < 0.001) and total time was significantly increased (*F* (5, 54) = 4.490, *P* < 0.01). These results revealed that the A*β*_1–42_-treated mice had obvious cognitive impairment. Moreover, the decrease of CR was reversed, respectively, by CF (180 mg/kg and 360 mg/kg) from second day to fourth day (*P* < 0.001 and *P* < 0.01; *P* < 0.05; *P* < 0.01 and *P* < 0.05 versus the model) and by donepezil treatment from the third to fourth day (*P* < 0.05, *P* < 0.05 versus the model). Besides, the increase of total time was shortened, respectively, by CF (180 mg/kg and 360 mg/kg) (*P* < 0.01 and *P* < 0.05 versus the model) and by donepezil treatment (*P* < 0.05 versus the model). In addition, the memory enhancing activity of CF was shown to be more potent than donepezil-treated group (0.65 mg/kg body weight, p.o.).

### 3.4. Morris Water Maze Test

**Figure 4:**
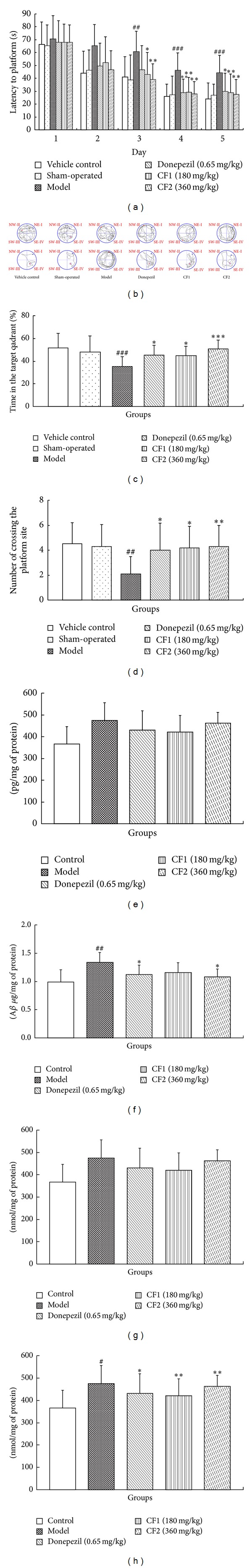
The Morris water maze test of mice treated with vehicle, A*β*_1–42_, A*β*_1–42_ + donepezil (0.65 mg/kg), A*β*_1–42_ + CF (180 mg/kg), and A*β*_1–42_ + CF (360 mg/kg), respectively. (a) Latencies to find a hidden platform in the water maze during five consecutive training days. (b) Search strategy of mice in the second trial on the second and fifth day. Traces show the swim path of all groups of mice. (c) The time spent in the quadrant where the platform was once placed within 60 s. (d) Number of crossings over the former platform location in the probe trial. (e) and (f) The effect of CF on *β*-secretase activity in the frontal cortex and accumulation of A*β*_1–42_ in hippocampus. (g) and (h) The effect of CF on acetylcholinesterase activity in the cortex and hippocampus of A*β*_1–42_-injected mice. Data are expressed as mean ± SD. ^#^ *P* < 0.05, ^##^ *P* < 0.01, and ^###^ *P* < 0.001 versus the control mice; *P < 0.05, **P < 0.01, and ***P < 0.001 versus the model group mice.

Morris water maze test was used to assess the spatial learning and memory ability of animals. As shown in Figure 4(a), the mean latency to find the platform declined progressively during the five training days. The model group mice markedly spent longer time in finding the platform than the vehicle control mice in all training days (d3,* F* (5, 234) = 2.707, *P* < 0.05; d4,* F* (5, 234) = 4.259, *P* < 0.01; d5,* F* (5, 234) = 3.232, *P* < 0.05). These results revealed that the model group mice had significant cognitive impairment. Moreover, CF1 and CF2 group mice significantly shortened the escape latency compared with the model group mice from the third to fifth day (42.92 ± 17.31 s versus 60.66 ± 15.75 s, *P* < 0.05; 29.32 ± 11.25 s versus 46.29 ± 13.48 s, *P* < 0.01; and 28.87 ± 14.56 s versus 44.36 ± 13.52 s, *P* < 0.01, resp.) and from the third day backwards (39.01 ± 19.92 versus 60.66 ± 15.75 s, *P* < 0.01; 27.78 ± 9.70 s versus 46.29 ± 13.48 s, *P* < 0.01; 27.55 ± 11.42 s versus 44.36 ± 13.52 s, *P* < 0.01), respectively. Meanwhile, donepezil (0.65 mg/kg) treatment reached the similar effect from the fourth to fifth day of training trials (28.77 ± 10.75 s versus 46.29 ± 13.48 s, *P* < 0.01; 29.83 ± 14.33 s versus 44.36 ± 13.52, *P* < 0.05, resp.).  Figure 4(b) illustrated the swim paths of mice in the second trial of the second and fifth day in this test. Mice tended to explore all four quadrants of the pool in the second day. Thereafter, they changed this search strategy. On the fifth day, the vehicle control mice swam in the direction of the platform; however, the model group mice took longer swimming paths. In the probe test (Figure 4(c)), the vehicle control mice spent longer time in the target quadrant than the model group mice (*F* (5, 54) = 3.376, *P* < 0.01). CF (180 and 360 mg/kg) and donepezil (0.65 mg/kg) treatment mice also took longer time in the target quadrant than the model group mice (*P* < 0.05, *P* < 0.001 and *P* < 0.05, resp.). It is more accurate to evaluate the effects of durg on impairment of spatial learning and memory through comparison of the number of crossing, as shown in Figure 4(d). The model group made fewer platform crossings than the vehicle control group (*F* (5, 54) = 2.628, *P* < 0.05) and the CF (180 and 360 mg/kg) and donepezil (0.65 mg/kg) treatments could increase the number of times of crossing over the platform site than the model group (*P* < 0.05, *P* < 0.01 and *P* < 0.05); however, CF2 had greater effect to reverse the deficit of spatial learning and memory induced by A*β*_1–42_ than donepezil treatments.

### 3.5. Effect of CF on Brain GSH-px Activities and MDA Contents

Table 3 summarized the antioxidant effect of CF in the A*β*_1–42_-injected mice. GSH-px is an important antioxidant enzyme involved in cellular protection against damage caused by oxygen-derived free radicals, by means of removing harmful peroxide metabolites and blocking lipid peroxidation chain reaction. A*β*_1–42_ suppressed GSH-px activity in the brain (*F* (4, 25) = 3.178, *P* < 0.05). However, CF2 (360 mg/kg) and donepezil groups displayed a significant elevation of GSH-px activity (*P* < 0.05 and *P* < 0.05 versus the model group, resp.). The MDA level in the brain of A*β*_1–42_-injected mice was higher than that of the vehicle control group (*F* (4, 25) = 4.439, *P* < 0.01). The increase was ameliorated by treatment of CF at doses of 180 or 360 mg/kg or donepezil (*P* < 0.01, *P* < 0.01 and *P* < 0.05 versus the A*β*_1–42_-treated group, resp.).

### 3.6. Inhibitory Effect of CF on *β*-Secretase Activity in the Frontal Cortex and Accumulation of A*β*_1–42_ in Hippocampus

The activity of *β*-secretase in the cortex of the model group was increased compared with vehicle control (Figure 4(e)). The administration of donepezil and CF lowered the increased activity of *β*-secretase induced by A*β*_1–42_ injection in cortex of mouse brain. However, it did not show any significant differences (*F* (4, 25) = 1.690, *P* > 0.05).

We further measured the A*β*_1–42_-levels in brain homogenate by ELISA. The ELISA results revealed that the A*β*_1–42_ levels were increased in A*β*_1–42_-treated group (*F* (4, 25) = 3.151, *P* < 0.05). Compared with the model group, the A*β*_1–42_ levels were significantly decreased in donepezil and CF2 treatment groups (*P* < 0.05, Figure 4(f)).

### 3.7. Inhibitory Effect of CF on AChE Activity in the Frontal Cortex and Hippocampus

As shown in Figure 4(g), the administration of CF or donepezil to A*β*_1–42_-injected mice produced no significant change on the activity of AchE in cortex (*F* (4, 25) = 1.533, *P* > 0.05). The activity of AchE in hippocampus was increased in A*β*_1–42_-injected mice and greater than those in vehicle control (*F* (5, 54) = 3.151, *P* < 0.05). However, the increased activity of AchE in hippocampus was significantly inhibited by the treatment with CF1 and CF2 (*P* < 0.01 and *P* < 0.01) (Figure 4(h)).

### 3.8. Effects of CF on Neurodegenerative Changes in the Frontal Cortex and Hippocampus

**Figure 5:**
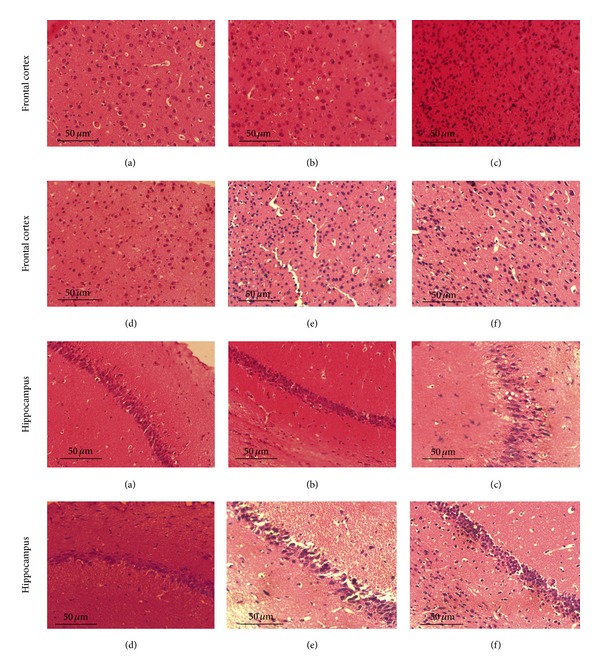
Effect of CF on neuronal degeneration in frontal cortex and hippocampus induced by A*β*_1–42_ treatment in mice (H&E staining, 400x). (a) Vehicle control group; (b) sham-operated group; (c) model group; (d) donepezil group (0.65 mg/kg); (e) CF1 group (180 mg/kg); (f) CF2 group (360 mg/kg).

As shown in Figure 5, (a) and (b) showed normal cells which are intact and there are less degeneration in normal cortex and hippocampus of mice. (c) A*β*_1–42_-induced neuron death was observed consistently in the cortex and hippocampus, as indicated by the appearance of pyknotic black neurons, karyorrhexis, and karyolysis with condensed nucleus. Figure 5(d) showed moderate damage to the cortex and hippocampus area pretreated with donepezil 0.65 mg/kg, indicated by presence of less number of degenerative cells compared to the model group. Figures 5(e) and 5(f) showed mild damage to the cortex and hippocampus area pretreated with CF (180 mg/kg, 360 mg/kg) indicated by presence of medium number of degenerative cells compared to the model group.

## 4. Discussion and Conclusion

In common parlance,* Alpinia oxyphylla* Miq. has been often touted as a drug hid in kitchen; therefore, it is not necessary to worry about the side effect of taking it. In terms of neuroprotection, it has been reported that ethanol extract of* Alpinia oxyphylla fructus* shows inhibition of tau protein phosphorylation in cell culture and the mechanism of sharp leaf galangal fruit extract can improve the spatial learning ability having business with its inhibiting ability on serum levels of cytokines [25]. For the preparation of the bioactive fraction of* Alpinia oxyphylla* which includes major sesquiterpenes with neuroprotective, antioxidant, or cognitive-enhancing activities, we chose chloroform fraction of ethanol extract of* Alpinia oxyphylla* Miq. However, screening of activities in vitro always lacks details of facts. To verify the potential of* Alpinia oxyphylla*, which was rich with neuroprotective or cognitive enhancing, we examined the protective effects of CF on cognitive impairment and neurotoxicity within the cortex and hippocampus in A*β*_1–42_-injected mice.

However, A*β* is transported bidirectionally across the blood-brain barrier (BBB), that is, both in brain-to-blood (efflux) and the blood-to-brain (influx) directions. Therefore, how to remove the toxic A*β*42 peptide through BBB safely and effectively is considered to be an effective method of the prevention and treatment of AD. Previous studies have shown the influence of traditional Chinese aromatic medicine between the structure and function of BBB. Modern pharmacological studies indicate that the permeability of BBB could be changed by traditional Chinese aromatic medicine in three ways. Firstly, it can develop potency by permeating the BBB reinlessly, secondly, it can protect the brain tissue by reducing the permeability of BBB, and thirdly, it can promote the opening of BBB, while its mechanism of action may be concerned with restraining the expression and function of P-glycoprotein. We supposed that sesquiterpenoids contained in CF may play the same role.

At present, there is no animal model can mimic all the cognitive, behavioral, biochemical, and histopathological abnormalities observed in patients with AD. Mice intracerebroventricularly injected with A*β*_1–42_ can mimic some cognitive deficits in AD, which is an economic and reliable model. Therefore, we first tested the memory enhancing effect of CF in mice treated with A*β*_1–42_. The cognitive-enhancing activity of CF was evaluated by Y-maze test, active avoidance test, and Morris water maze test. CF mitigated the impairment of recognition induced by A*β* in mice. The treatment of CF more effectively improved the deficit in long term memory than in short term memory. Oral treatment with CF also exhibited a mitigation of memory deficit in mice induced by i.c.v. injection of A*β* on escape latency, time spent in target quadrant, and cross-platform times in Morris water maze test. The treatment with CF decreased the latency to a shorter level than that of nontreated normal control. In addition, cognitive-enhancing effect of CF was found to be much more effective than that of donepezil. Besides, the same effect was observed in active avoidance test; the data showed that CF exhibited significant influence on condition avoidance response (CR) and total time (TT). The results indicate that CF has the potential to ameliorate cognitive deficits induced by A*β*_1–42_.

In spite of conflicting hypothesis on the pathological progress in AD patients, it is now generally recognized that the accumulated amyloid plaque in the brain is characteristic and diagnostic features of AD. *β*-secretase initiates the cleavage of *β*-amyloid precursor protein (APP) leading to the production of amyloid-*β* (A*β*); the gathering of A*β* is the main cause of senile plaque in the brain of AD patient, causes neuron damage and the hypofunction of cognition, and works as the center and cogallery in the pathogenesis of AD. The suppressant's partial inhibition of *β*-secretase can decrease the production of A*β* and get a manifesting therapeutic effect. Further study on the way BACE inhibitors work has favourable perspective in the treatment of AD. Our data showed that the activities of BACE1 increased by A*β* injection in the cortex of mouse brain were significantly inhibited by CF. Accumulation of deposits of A*β* is one of the classical neuropathological hallmarks in AD and may be the primary event in the pathogenesis of AD. In accordance with the reduction in BACE1 activity by CF, the aggregation of A*β* in the hippocampus of A*β*-injected mouse brain was found to be markedly reduced by CF by enzyme-linked immunosorbent assay (ELISA).

Growing data from experimental models and human brain studies suggest that oxidative stress, inflammation, and apoptotic cell death induced by A*β* play important roles in neuronal degeneration in AD [26]. Each of these factors can act independently or collectively damage neurons and disturb cognitive processes.

The oxidative stress hypothesis of AD suggests that the aggregation of A*β* on the membrane generates reactive oxygen species (ROS), resulting in protein oxidation, lipid peroxidation, cellular dysfunction, and subsequent neuronal death [27]. It may be one of the first pathogenic events during disease progression. In the A*β*_1–42_-induced model mice, the level of MDA (a well-known indicator of lipid peroxidation) increased, and activities of GSH-px (the line of the antioxidant defense systems) decreased. GSH-px scavenges ROS by directly reacting with it and prevents H_2_O_2_-induced hydroxyl radical formation. GSH-px level parallels the antioxidant defense capacity and is a first indicator for oxidative stress in the brain [28]. Thus, the increase in activities of antioxidant defense systems is considered to be beneficial in the event of oxidative stress. The results indicated that an increase of oxidative stress occurred in the brain of A*β*_1–42_-treated mice. After the administration of CF, the decreased activities of GSH-px, as well as the increased level of MDA, were restored in the brain. The possible mechanisms of CF are largely related to its antioxidant and other scavenging properties, for example, over nitric oxide (NO), possibly, through downregulation of NOS activity in brain. Previous phytochemical investigation on this plant revealed oxyphyllanene A, teuhetenone A, oxyphyllol B, and nootkatone with inhibitory activity against NO production in LPS and IFN-c-induced RAW 264.7 macrophages, which showed that these compounds exhibited potent activities of NO inhibitory and neuroprotection [12]. Besides, it was reported that Alpinia protocatechuic acid protects against oxidative damage in vitro and reduces oxidative stress in vivo [14]. Therefore, it is undoubtedly that CF could effectively attenuate oxidative stress induced by A*β*.

Microglia, the resident macrophages in the central nervous system, can be activated by A*β*. Oligo-A*β*_1–42_ is always likely to damage neurons through the microglia-inflammation response. Activated microglia not only can eliminate A*β* but also can release inflammatory factors, which play a critical role in AD pathology. Recently reports strongly suggest that regulating microglia function may be a promising therapeutic approach to AD. From histological evidence of tissue sections, we observed that the model group mice showed significant microglial cell hyperplasia, neuronal disorder, and degeneration in the frontal cortex and hippocampus, while in CF (180 mg/kg and 360 mg/kg) treated group the microglia activation, neuronal acidophilia degeneration, and nuclear condensation decreased, resulting in homogeneous morphology of neuronal cellular.

It has been demonstrated that impairments in learning, memory, and behavior observed in AD patients are associated with the cholinergic hypofunction. Neuronal loss in the basal forebrain particularly within the septohippocampal acetylcholinergic systems involved in learning and memory processes constitutes a pathological hallmark of AD. The mechanism of aggregated A*β*_1–42_ peptide-induced increase of AChE activity was related with two pathways: (1) indirect action-increase intracellular Ca^2+^ and free radical, which are symbols of cellular damage and (2) direct action-change conformation of AChE. Recently, it has been reported that ethanolic extracts of* Alpinia oxyphylla* Miq. showed most potent AChE inhibitory activities at the concentration of 0.1 mg/mL, with 44.49% inhibition among 48 traditional Chinese medicinal herbs [29]. In our research, administration of CF significantly showed greater inhibition activity of AChE than donepezil known as the most common prescribed AChE inhibitor in hippocampus of A*β*-injected mouse. It may be related to acetylcholinesterase inhibition of nootkatone [30].

From the above results, it could be deduced that cognitive-enhancing activity of CF might result in part from the inhibition on the accumulation of A*β* and the BACE1 and AChE and from the reduction in ROS by recovering the antioxidative defense system. These results suggested that chloroform extract of* Alpinia oxyphylla* containing bioactive sesquiterpenes, such as oxyphyllanene A, teuhetenone A, oxyphyllol B, and nootkatone, might offer a useful therapeutic choice in either the prevention or the treatment of Alzheimer's disease.

## Figures and Tables

**Table 1:** Retention times, MS, and MS/MS^2^ fragmentation patterns of the substances in chloroform extract of *Alpinia oxyphylla. *

Peak	*T*_*R*_ (min)	MS^+^ (*m*/*z*)	Major fragments (positive ion mode) (*m*/*z*)	Tentative identification
1	1.08	192.1234	175.1[M+H-H_2_O]^+^→ 151.1[M+H-CH_2_CO]^+^ → 133.1[M+H-CH_2_CO-H_2_O]^+^	Oxyphyllanene A

2	1.22	153.8901	110.8[M+H-CO_2_]^+^	Protocatechuic acid

3	3.47	220.1544	177.1 → 133.1	Unknown

4	3.96	252.1821	235.1[M+H-H_2_O]^+^→ 206.1[M-3CH_3_]^+^ → 189.1[M+H-3CH_3_-H_2_O]^+^→ 177.1 [M-C_3_H_5_-2OH]^+^→ 107.1	11S-nootkatone-11,12-diol

5	4.06	252.1797	235.1[M+H-H_2_O]^+^→ 206.1[M-3CH_3_]^+^ → 189.1[M+H-3CH_3_-H_2_O]^+^→ 177.1 [M-C_3_H_5_-2OH]^+^→ 107.1	11R-nootkatone-11,12-diol

6	6.62	194.1307	177.1[M+H-H_2_O]^+^→ 149.1[M+H-H_2_O-CO]^+^→ 135.1 [M+H-H_2_O-CO-CH_3_]^+^→ 120.1[M+H-H_2_O-CO-2CH_3_]^+^	Teuhetenone A

7	8.10	194.1389	177.1[M+H-H_2_O]^+^→ 161.1[M+H-H_2_O-CH_2_]^+^→ 137.1[M+H-H_2_O-CO-CH_2_]^+^	Teuhetenone B

8	10.18	220.1547	203.1 → 159.1	Unknown

9	14.21	222.1705	180.1 → 138.1	Unknown

10	15.82	216.1595	179.1 → 137.1	Unknown

11	16.48	234.1704	217.2[M+H-H_2_O]^+^→ 207.2[M+H-CO]^+^→ 203.2[M+H-H_2_O-CH_2_]^+^ → 189.2[M+H-H_2_O-2CH_2_]^+^	Oxyphyllol B

12	20.47	218.1671	176.1[M+H-C_3_H_6_]^+^ → 163.1[M+H-C_3_H_5_-CH]^+^ → 149.1[M+H-C_3_H_5_-CH-CH_2_]^+^ → 135.1[M+H-C_3_H_5_-CH-CO]^+^	Nootkatone

13	24.53			Dibutyl phthalate

**Table 2:** Effect of CF on condition avoidance response and total time induced by A*β*_1–42_ in mice.

Group	Condition avoidance response/%				Total time/s
	1	2	3	4 (d)	
Control	46.0 ± 29.3	59.3 ± 20.0	86.0 ± 9.7	86.0 ± 9.7	1542 ± 102
Sham-operated	46.7 ± 30.5	74.0 ± 21.4	85.3 ± 14.3	85.3 ± 15.6	1482 ± 104
Model	34.0 ± 18.9	37.3 ± 20.4^#^	49.3 ± 28.5^###^	51.4 ± 23.3^###^	1683 ± 70^##^
Donepezil 0.65 mg/kg	42.7 ± 34.6	48.0 ± 26.9	70.0 ± 29.2*	68.7 ± 23.5*	1586 ± 115*
CF1 180 mg/kg	48.7 ± 32.9	71.3 ± 18.6***	70.0 ± 24.0*	74.7 ± 16.9**	1544 ± 121**
CF2 360 mg/kg	42.6 ± 27.5	67.3 ± 23.6**	68.0 ± 22.0	68.7 ± 22.4*	1585 ± 77*

Results are expressed as mean ± SD, *n* = 60 (10 mice each group), ^#^ *P* < 0.05, ^##^ *P* < 0.01, and ^###^ *P* < 0.001 versus the control mice; *P < 0.05, **P < 0.01, and ***P < 0.001 versus the model group mice.

**Table 3:** Effect of CF on brain tissue GSH-px activities and MDA contents.

Group	GSH-px (U/mg protein)	MDA (nmol/mg protein)
Control	4.33 ± 0.49	1.42 ± 0.30
Model	3.44 ± 0.39^#^	1.93 ± 0.47^##^
Donepezil 0.65 mg/kg	4.34 ± 0.94∗	1.56 ± 0.40∗
CF 180 mg/kg	3.77 ± 0.77	1.31 ± 0.23∗∗
CF 360 mg/kg	4.54 ± 0.98∗	1.25 ± 0.21∗∗

Results are expressed as mean ± SD,  *n* = 6,  ^#^ *P* < 0.05,  ^##^ *P* < 0.01 versus the control mice;  *P < 0.05,  **P < 0.01 versus the model group mice.
